# Current methods for detecting and assessing HIV-1 antibody resistance

**DOI:** 10.3389/fimmu.2024.1443377

**Published:** 2025-01-06

**Authors:** Stanley Odidika, Martin Pirkl, Thomas Lengauer, Philipp Schommers

**Affiliations:** ^1^ Department I of Internal Medicine, Faculty of Medicine and University Hospital Cologne, University of Cologne, Cologne, Germany; ^2^ Center for Molecular Medicine Cologne (CMMC), Cologne, Germany; ^3^ German Center for Infection Research (DZIF), Partner Site Cologne-Bonn, Cologne, Germany; ^4^ Institute of Virology, Faculty of Medicine and University Hospital Cologne, University of Cologne, Cologne, Germany; ^5^ Max Planck Institute for Informatics and Saarland Informatics Campus, Saarbrücken, Germany

**Keywords:** HIV, antibody, mutation, aids, bNAbs, broadly neutralizing antibodies

## Abstract

Antiretroviral therapy is the standard treatment for HIV, but it requires daily use and can cause side effects. Despite being available for decades, there are still 1.5 million new infections and 700,000 deaths each year, highlighting the need for better therapies. Broadly neutralizing antibodies (bNAbs), which are highly active against HIV-1, represent a promising new approach and clinical trials have demonstrated the potential of bNAbs in the treatment and prevention of HIV-1 infection. However, HIV-1 antibody resistance (HIVAR) due to variants in the HIV-1 envelope glycoproteins (HIV-1 Env) is not well understood yet and poses a critical problem for the clinical use of bNAbs in treatment. HIVAR also plays an important role in the future development of an HIV-1 vaccine, which will require elicitation of bNAbs to which the circulating strains are sensitive. In recent years, a variety of methods have been developed to detect, characterize and predict HIVAR. Structural analysis of antibody-HIV-1 Env complexes has provided insight into viral residues critical for neutralization, while testing of viruses for antibody susceptibility has verified the impact of some of these residues. In addition, *in vitro* viral neutralization and adaption assays have shaped our understanding of bNAb susceptibility based on the envelope sequence. Furthermore, *in vivo* studies in animal models have revealed the rapid emergence of escape variants to mono- or combined bNAb treatments. Finally, similar variants were found in the first clinical trials testing bNAbs for the treatment of HIV-1-infected patients. These structural, *in vitro*, *in vivo* and clinical studies have led to the identification and validation of HIVAR for almost all available bNAbs. However, defined assays for the detection of HIVAR in patients are still lacking and for some novel, highly potent and broad-spectrum bNAbs, HIVAR have not been clearly defined. Here, we review currently available approaches for the detection, characterization and prediction of HIVAR.

## Introduction

Human immunodeficiency virus (HIV) remains a global health challenge, with an estimated 38 million people living with the virus worldwide (1). Although antiretroviral therapy (ART) has significantly improved the management of HIV, there is still a need for more effective approaches for treatment and prevention. Antiretroviral therapy (ART) can effectively suppress HIV-1 replication and disease progression to AIDS but requires lifelong daily medication that comes with long-term toxicities. Although effective ART has been available for about 20 years now, there are still 1.5 million new HIV infections and 700,000 AIDS-related deaths each year (1). Antiretroviral therapy has not only significantly improved the health and well-being of individual HIV-1 infected patients in recent years but has also played a pivotal role in effectively combating the HIV pandemic on a global scale. However, the ongoing pandemic, the lack of a vaccine and the fact that there is still no cure for HIV-1 underscore the urgent need for next-generation therapies to treat, prevent or even cure HIV-1 infection.

**Figure 1:**
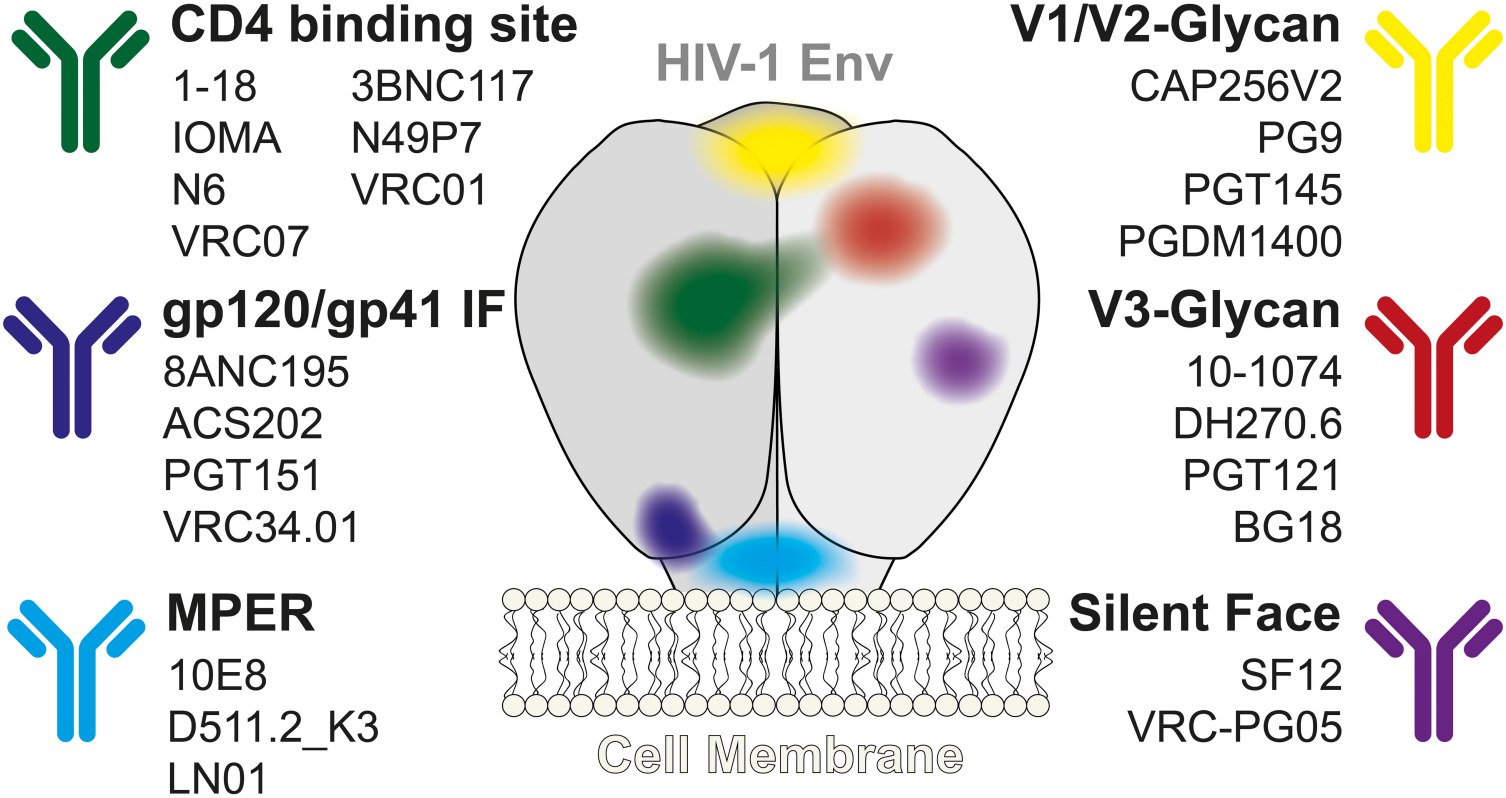
HIV-1 reactive Broadly Neutralizing Antibodies. Overview of a selection of broadly neutralizing antibodies against HIV-1 and their corresponding epitopes on the HIV-1 Env protein. IF, Interface; HIV-1 Env, HIV-1 envelope glycoprotein; MPER, membrane-proximal external region.

Only a few HIV-1-infected individuals (1-5%) develop exceptionally high titers of HIV-1 neutralizing serum activity. From these so-called ‘elite neutralizers’ broadly neutralizing antibodies (bNAbs) targeting the HIV-1 envelope glycoprotein (HIV-1 Env) have been isolated from several B cell lineages. This resulted in a wide range of bNAbs with a coverage (defined by the number of HIV strains neutralized) of up to 100% and high potency (defined by the inhibitory concentration (IC) that neutralizes the virus). Several bNAbs targeting different epitopes of the HIV-1 Env have been well characterized, such as 3BNC117, VRC01, VRC07-523LS, 1-18, N6, IOMA and N49P7 targeting the CD4-binding site (CD4bs) (2–8), CAP256V2, PG9, PGDM1400 and PGT145 specific for the V1/V2-glycans (9–12), 10-1074, PGT121, DH270.6 and BG18 that are specific for V3-glycan (13–15), 10E8, DH511.2_K3 and LN01 specific for MPER (16–18), 8ANC195, VRC34.01, ACS202 and PGT151 which target the gp120/gp41 interface region (3, 19–21) and SF12 and VRC-PG05 which target the so-called ‘silent face’ region on HIV-1 Env (**Figure 1**) (22, 23). In addition to their antiviral activity, these bNAbs have several advantages over currently available ART. HIV-1 reactive bNAbs have a half-life of 14-21 days which can even be prolonged by FC domain modifications, they contribute to the active clearance of the pathogen through the engagement of innate effector responses (FC-mediated effects such as antibody-dependent cellular cytotoxicity (ADCC)), and they enhance immunity against HIV-1 (24–27).

The available evidence from *in vivo* experiments in non-human primates and humanized mice strongly supports the promising role of bNAbs in both the future prevention and treatment of HIV-1. HIV-1 reactive bNAbs have shown potent antiviral activity in viremic HIV-1 infected humanized mice as well as in simian-human immunodeficiency virus (SHIV) infected non-human primates (28, 29). In addition, bNAbs have been shown to protect against viral challenge in these animal models (30–33). HIV-1 reactive bNAbs have already been tested in many clinical trials in recent years, where they demonstrated safe suppression of viremia and delay of viral rebound after ART interruption in HIV-1-infected individuals (34–36). Having established their efficacy in patients, researchers then tested on to test combinations of bNAbs, targeting different epitopes, and found improved suppression of plasma viremia and a prolonged time to viral rebound after an analytical treatment interruption compared to bNAb monotherapies (37, 38). In addition, a large multicenter study showed that intravenous infusion of the bNAb VRC01 can protect humans against infection with VRC01-sensitive HIV-1 isolates (39, 40). This study demonstrated that bNAbs can prevent infection by bNAb-sensitive strains in humans and that the bNAb serum titer can be a useful indicator of protection. Therefore, the induction of bNAbs has become an important goal for vaccine development (41, 42). Overall, antibodies have already been groundbreaking in the treatment of autoimmune diseases and cancer. And with the identification of new, highly potent HIV-1 broadly neutralizing antibodies (bNAbs) in recent years and increasing evidence of their beneficial properties, bNAbs will also play a key role in future HIV-1 treatment and prevention strategies.

However, as with any drug against HIV-1, viral resistance, and escape are formidable challenges for future approaches to HIV-1 treatment, prevention and cure involving bNAbs (39, 40, 43). RNA viruses, such as HIV-1, are characterized by exceptionally high rates of spontaneous mutations (44–46). This results in a large diversity of viral strains and their ability to rapidly escape humoral or cellular immune pressure by selecting viral variants with amino acid mutations that block detection or binding by immune cells or antibodies (47). While numerous studies have demonstrated the extraordinary potential of bNAbs and bNAb-inducing vaccines, HIVARs that are pre-existed in patients or develop *de novo* during treatment have significantly impaired bNAb activity.

Since the introduction of effective antiretroviral drugs about 30 years ago, viral resistance has played a critical role in selecting the appropriate treatment for each individual. HIV drug resistance to conventional antiretroviral treatment (ART) can be either acquired or transmitted (48). Acquired HIV drug resistance occurs after initial effective suppression of wild-type HIV variants during ART; some mutated and drug-resistant strains are selected during the viral replication process, leading to viral rebound. Transmitted drug resistance occurs when drug-naive HIV-infected patients are initially infected with drug-resistant variants, resulting in an ineffective response to ART. During the introduction of ART in the 1990s, studies showed that the combination of ARTs targeting different steps of the HIV-1 life cycle could prevent the rapid emergence of escape variants (49, 50). Today, combination regimens of two to three antiviral drugs are the gold standard for HIV-1 treatment (51). Compared to ART resistance, HIVARs are much more common in patients, most likely as a result of the humoral immune response that naturally targets the HIV-1 Env after infection. The large number of patients harboring HIVARs and the rapid emergence of viral escape variants after monotherapies forced the field to develop better strategies to overcome viral escape. And similar to the development of today’s effective combination ART, researchers began to use multiple bNAbs to increase the percentage of strains covered (37, 38). In addition, newer, broader and more potent bNAbs have been identified in recent years, some of which have even been able to limit viral escape and/or resistance mechanisms *in vivo* (5). Although HIVARs are a critical issue for the future use of bNAbs in the clinic, only a few studies have specifically focused on a better identification, characterization and understanding of HIVARs and their evolution.

**Figure 2:**
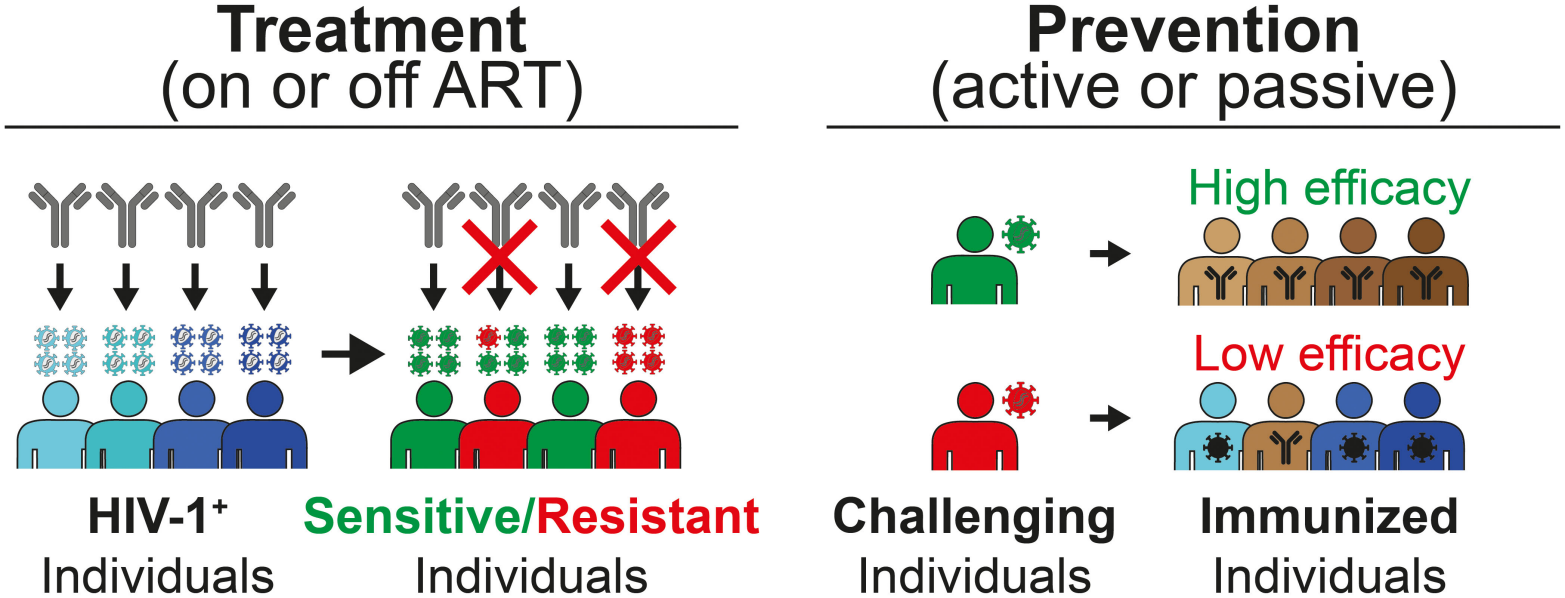
Scenarios that require detection of HIVARs. Left panel: HIV-1 infected patients need to be screened for bNAb susceptibility prior to treatment. For this purpose, circulating viruses or proviral sequences would have to be tested for HIV-1 antibody resistance (HIVAR). Right panel: Pre-existing HIVAR may hinder the future use of bNAbs for prevention, either by passive administration or by bNAb-eliciting immunogens. Pre-existing HIVARs (red patient) in the population that challenge the immunized individuals can significantly hamper the efficacy of and, more importantly, the efficacy of bNAb-inducing vaccines that will only protect against viral strains that are sensitive to the induced bNAb. ART, antiretroviral therapy.

Most importantly, the field currently lacks thoroughly clinically validated assays that can detect HIVARs in circulating and/or proviral strains. Novel and improved methods and assays are urgently needed to detect HIVARs in patients in a feasible and timely manner, as HIVARs may significantly interfere with the activity of bNAbs and thus significantly impact the efficacy of future bNAb-mediated treatment and prevention approaches. Scenarios in which the detection of HIVARs would be required include, but are not limited to, the detection of HIVARs: i) in circulating strains of naive patients prior to initiation of bNAb treatment, ii) in replication-competent proviruses of virologically suppressed patients prior to switching from classical antiretroviral treatment to bNAb treatment, and iii) in HIV-1 infected populations for the development of novel vaccines that elicit bNAbs capable of protecting against prevalent strains (**Figure 2**).

In summary, while bNAbs hold great promise for future HIV-1 treatment and prevention approaches, *de novo* and pre-existing HIV-1 antibody resistance (HIVAR) pose significant barriers to the clinical use of bNAbs and potential future bNAb-inducing vaccines. Therefore, the field needs a deeper understanding of the impact of envelope resistance mutations on bNAb activity. In addition, assays for the rapid detection of HIVAR in circulating patient viruses and proviruses are urgently needed. In this review, we discuss the recent advances in the identification and characterization of HIVAR and their potential impact on the future clinical use of bNAbs. As the majority of patients are now on effective antiretroviral therapy (ART), we will also focus on the identification of HIVAR in the proviral reservoir of patients. We also provide insights into computational approaches to predict the sensitivity of HIV variants to bNAbs for clinical settings.

## Structural analyses can inform on residues critical for sensitivity to viral neutralization

The HIV Env trimer consists of three heterodimers, the surface gp120 and the transmembrane gp41, formed after cleavage of gp160 by furin (52–55). After infection with HIV-1, the vast majority of patients develop antibodies that bind to HIV-1 Env, while only a small subset develop antibodies that are neutralizing, as the process of inducing such broadly neutralizing antibodies (bNAbs) faces significant challenges. The Env protein has robust defenses that impede the development of bNAbs (56). The trimer exhibits conformational dynamics, considerable sequence variability - especially in regions accessible to antibodies on the envelope - and is sparsely distributed throughout viral particles (57). In addition, it is highly glycosylated, with tightly packed glycans obscuring much of the underlying protein surface (58). Immunological decoys, such as non-functional envelope proteins like gp41 stumps, monomeric forms of gp120, and non-functional protomers (e.g., uncleaved trimers) that expose non-neutralizing epitopes typically hidden in the trimer, add another layer of complexity (59). HIV-1 Env tends to be inherently unstable, transitioning into non-functional configurations over time (59, 60). In certain cases, gp120 subunits can dissociate from gp41, leaving in the latter remaining as stumps on the virion surface (58, 59). An alternative view is that the spike is not inherently unstable but originates from the plasma membrane of the infected cell in an early form, exposing immunological epitopes that are not present in the later mature HIV-1env (61). This instability of HIV-1 Env required various sequence modifications, fixations by truncated CD4 and stabilizations with antibodies in order to solve the first gp120 structure by X-ray crystallography (62).

Since its initial in-depth structural characterization, further analysis of bNAb-HIV-1 Env complexes has provided information on residues that may be critical for binding and/or neutralization (62). While imaging techniques have evolved over time, trimers mimicking native-HIV-1 Env have also contributed significantly to the structural understanding of bNAb-HIV-1 Env interactions. For example, the introduction of SOSIP trimers, named after the mutations that result in a stable and soluble recombinant HIV-1 Env trimer, has contributed tremendously to the understanding of bNAbs binding to vulnerable sites on the native HIV-1 Env trimer (63). Since then, several stable trimers have been generated using isolates different from BG505, and also novel approaches to stabilize trimers that do not use the SOSIP mutation have been developed to better characterize the binding kinetics of bNAbs to native-like Env trimers (64–66).

Following the identification of a novel bNAb with an unknown binding site, a competition ELISA assay, in which the novel bNAb competes with antibodies with known targets for binding to HIV-1 Env, can often provide information about the target site of the particular bNAb (5, 9, 13, 16, 23, 67). However, only structural data, as described above, can provide information on the antibody-envelope interaction at the atomic level. Thus, cryo-EM data of bNAbs bound to either HIV-1 gp120, gp140 or SOSIP trimers were used to identify the exact binding sites of each antibody (3, 5, 6, 18, 68–71). Subsequently, several studies mutated the amino acids on the envelope that were found to be in contact with the tested antibody in order to confirm their relevance in binding and/or neutralization. And in several cases, high-resolution HIV-1 Env-antibody complexes suggested contact sites for bNAbs that did not appear to be critical *in vitro* for neutralization when these amino acids were mutated in pseudoviruses (5, 16, 23). Thus, structural analysis alone cannot discriminate between the importance of an antibody-envelope amino acid interaction and may not always provide a complete understanding of the functional significance of specific amino acid residues within the antibody-epitope interaction. While structural studies reveal the spatial arrangement of antibody and epitope residues at atomic resolution, they do not account for potential allosteric effects. Mutations at sites distal to the direct antibody-epitope interface can induce conformational changes that alter the dynamics of the epitope and indirectly affect antibody binding or neutralization. Such effects may modulate the accessibility or stability of the binding site without disrupting the direct contacts identified by structural analysis. This highlights the need for functional assays to evaluate the broader impact of specific residues on antibody binding and neutralization efficacy. In summary, structural data alone cannot provide a complete picture of the amino acid residues that are critical for antibody activity, and additional *in vitro* and *in vivo* analyses are needed to provide a more nuanced understanding of antibody functionality.

## Env conformational flexibility impacts viral resistance to bNAbs

Recent studies highlight different methods to study Env conformational flexibility and antibody resistance, shedding light on the mechanisms underlying Env conformational flexibility and resistance to bNAbs. Single-molecule Förster resonance energy transfer (smFRET) is one of the most frequently used techniques to study conformational changes and dynamics of biomolecules. The smFRET approach has provided insights into the conformational transitions between different Env states (72–74). By analyzing conformations such as the pre-triggered (closed) (state 1), intermediate (state 2), and fully CD4-bound (state 3) states, smFRET analysis links HIV-1 Env flexibility to differences in antibody sensitivity. The identification of state 2 as a functional intermediate provides a clearer picture of the stepwise conformational changes required for HIV-1 entry (74). This intermediate state serves as a critical checkpoint in the HIV fusion process. Additional binding studies with bNAbs revealed minor differences in the antigenic profiles between states 1 and 2, while a marked disparity was observed between states 1 and 3, with predicted root mean square deviations (RMSDs) between epitopes in state 1 and states 2 and 3 of ~2 Å and >30 Å, respectively (73). Interestingly, HIV-1 Env mutants enriched in state 2 show increased sensitivity to CD4 mimetics but greater resistance to certain bNAbs (74). Another study using the smFRET approach showed that CRF01_AE Env adopts an intermediate state 2 conformation, making it inherently more susceptible to ADCC than other subtypes such as clade B (72).

In support of the smFRET approach, further work structurally characterized the interaction between HIV Env trimers and CD4 molecules on target cells using cryo-electron tomography (cryo-ET) (75). Binding of HIV Env trimers to the CD4 molecule restructures Env from a closed to a partially open conformational state. This stepwise process involves first binding of the Env trimer to a single CD4 receptor on target cells and subsequent binding to a second and third CD4 molecule (75–77). Thus, Env trimers require binding to multiple CD4 molecules for effective viral entry. Cryo-EM studies of unliganded 1059-SOSIP Env further revealed asymmetry, increased flexibility, and “breathing” motions, supporting previous molecular simulations and structural hypotheses (78). The authors showed that some transmitted/founder HIV-1 strains harbor Env glycoproteins that exhibit incompletely closed conformations. This incompletely closed conformation of the HIV Env trimer allows the exposure of internal epitopes, which affects the susceptibility of Envs to bNAbs. For example, analysis of intermediate state Envs revealed different resistance profiles to VRC01-like CD4 binding site (CD4bs) bNAbs and V3 glycan-targeting bNAbs such as PGT121. Further analysis attributed the breadth and potency of bNAb N6 to its efficacy in neutralizing Env trimers with tightly closed and intermediate conformational states. Despite these interesting findings, these studies are limited to SOSIP and require further investigation of a substantial number of mutant HIV strains by cryo-EM (78).

In conclusion, studies using smFRET and Cryo-ET have revealed the critical role of intermediate Env conformations in HIV-1 entry and bNAb resistance, with state 2 serving as a key checkpoint. These findings highlight the link between HIV-1 Env flexibility and antibody susceptibility, although further research in diverse HIV-1 strains is needed to fully understand the implications for vaccine and therapeutic development.

## *In vitro* approaches to detect and characterize HIV-1 antibody resistance

The humoral response exerts selective pressure on the viral envelope, resulting in changes in the amino acid sequence of the HIV-1 envelope genome (HIV-1_env_) and thus increasing resistance to neutralizing antibodies over time in the HIV-1-positive population (43, 79). Moreover, at the individual patient level, the detection of mutations in HIV-1_env_ that render a virus resistant to a given humoral immune response is of great importance. Certain single or combined amino acid mutations in HIV-1 Env can confer resistance to bNAbs. Therefore, knowledge of these mutations is crucial for the future clinical use of bNAbs in novel prevention and treatment strategies (80, 81).

A commonly used approach to test single or combined HIV-1_env_ amino acid mutations for their impact on antibody sensitivity is the use of mutant pseudoviruses. In this so-called phenotypic resistance test, which is analogous to the phenotypic testing of viral resistance to classical antiviral drugs, a backbone pseudovirus is used and amino acid mutations are induced by site-directed mutagenesis or similar methods. Subsequently, the effect of a specific mutation in the HIV-1_env_ on its neutralizing activity can be assessed using *in vitro* assays such as the TZM-bl cell neutralization assay (82). This assay uses HeLa-derived TZM-bl cells expressing CD4, CXCR4, CCR5, and tat-reporter genes for luciferase and β-galactosidase (83, 84). These cells can be infected with HIV-1 Env pseudotyped viruses and infection rates can be determined using the luciferase or β-galactosidase reporter function. Thus, the neutralizing activity of polyclonal or monoclonal antibodies can be assessed by comparing the infection rates with and without the presence of the respective antibodies (85).

However, this assay is not without limitations. The concept of this approach only works if the critical amino acid sites of a given antibody are already known. This knowledge must be obtained from previous assays, such as the structural analyses mentioned above, similar data from bNAbs of the same class, or *in vivo* experiments in which escape mutations have been detected after treatment. However, in some cases, e.g. where structural assays can only indicate the contacted amino acids, it is still unclear whether only some of the other possible amino acids at the site may lead to impaired neutralization. Most groups use alanine scans for this approach, mutating all residues of interest to alanine before testing the mutant HIV-1 Env for antibody sensitivity (86). While alanine is chosen for its simple structure and non-polarity, there may be residues where this amino acid still retains bNAbs activity while others may render the virus resistant (17, 87). This approach is laborious and, while it may be possible to induce single amino acid mutations at different sites, it is impossible to test all possible combinations of mutations since even if only 3-4 sites are thought to be critical, the potential number of possible mutations easily goes into the hundreds or thousands. Moreover, some mutations induced *in vitro* may prove detrimental to the viral fitness *in vivo*, preventing their emergence.

Finally, studies have reported that the use of HIV-1 Env pseudotyped viruses may overestimate the breadth and potency of bNAbs (88–90). To circumvent this problem, the comparison of *in vitro* data using Env pseudotyped viruses and primary HIV-1 isolates is crucial before making clinical trial decisions. In particular, for the screening of a patient’s bNAb sensitivity prior to treatment trials, the use of viral outgrowth cultures has been shown to be feasible, effective, and to be able to predict a patient’s clinical response to bNAb treatment. However, the process of T-cell activation and culture can take up to several weeks, and some patients that were found to have a sensitive viral outgrowth culture still had no virologic response to the bNAb treatment (35–37). The latter may be explained by the finding that these cultures do not reflect the large diversity of replication-competent proviruses in a patient’s viral reservoir (91).

## *In vitro* viral evolution and adaption assays

To test viruses for possible escape mutations without any prior knowledge of the antibody being tested, novel approaches such as the Mutational Antigenic Profiling (MAP) assay have been developed in recent years (92, 93). This assay allows for the highlighting of viral escape pathways by generating 19 amino acid mutations at 670 different HIV-1 Env sites, resulting in 12,730 individual HIV-1 Env mutations. The corresponding libraries of viral quasispecies are then incubated with human polyclonal IgGs or bNAbs. Humoral immune pressure then selects for a swarm of resistant quasispecies, which subsequently allows identification of fractions of mutant viruses that survived antibody neutralization by each bNAb by deep-sequencing the HIV-1_env_ of strains that were able to infect cells in viral outgrowth assays in the presence of antibodies. The authors further revealed different mechanisms of viral resistance to bNAbs: (i) viral escapes to clonal variants of bNAbs are quite different, despite shared contact sites; (ii) mutations in Env trimers induce alteration of electrostatic charges and thus mediate bNAb resistance; (iii) alteration of glycosylation sites near or distal to epitopes of CD4bs bNAbs affects their neutralizing activities (92–94). Of note, viral resistance to neutralizing antibodies may develop more rapidly *in vivo* than *in vitro* (95) (92). Although antibody resistance induced by point mutations in this experimental approach correlates with those in human studies (35–37, 96), the majority of these mutations may not occur naturally in HIV-1-infected individuals (97), as they are detrimental to the viral replication capacity, the so-called ‘viral fitness’. Another caveat of this assay is its inability to determine the effect of combined mutations on the neutralization sensitivity to a given bNAb. The introduction of multiple mutations in well-defined epitopes on HIV-1 Env in a soft randomization approach has been shown to rapidly identify viral antibody escape (98). With soft randomization, HIV-1 Env libraries with multiple mutations in a single Env can be generated to investigate how diverse mutations in a single HIV Env affect viral sensitivity and fitness. Compared to MAP, soft randomization is relatively inexpensive and less labor-intensive for library generation. Focusing on bNAbs targeting the conserved CD4 binding sites on HIV-1 Env, Otsuka, and colleagues reported that viral escape from a single bNAb may increase viral resistance to other bNAbs in the same class (98). Thus, the design of combination bNAb therapies or multispecific bNAbs targeting different epitopes is critical to combat viral escape. Recently, Radford et al. developed the deep mutational scanning (DMS) approach, which can also determine the escape to polyclonal or monoclonal antibodies that may only be mediated by combinations of mutations (99).

In conclusion, testing large panels of pseudoviruses for their neutralization sensitivity, using pseudoviruses with specific amino acid mutations and developing novel assays such as the MAP and DMS assays are helpful in understanding viral escape pathways.

## Bioinformatic prediction of viral drug resistance

Computational methods have previously been successfully used for prediction in various settings. For example, several tools for predicting HIV drug resistance and treatment success have been implemented and are available as web applications. The Stanford Algorithm (https://hivdb.stanford.edu/hivdb/by-patterns/ (100)) and HIV-GRADE (https://www.hiv-grade.de/cms/grade/ (101)) are rules-based, which is one of the simplest approaches. The idea is to define escape mutations, also called resistance-associated mutations (RAMs), and relevant rules for predicting a virus as resistant or susceptible to a drug. In the of HIV-1 drug resistance, the mutations are most often found in the viral protease, reverse transcriptase or integrase genes for each drug or class of drugs that target that region. The Stanford algorithm uses a list of RAMs for each drug class with associated resistance scores ranging from -15 to 60 (102). The scores of all observed mutations are summed and the final score places the virus in one of five categories from susceptible (<10) to highly resistant (>60) to the drug.

**Figure 3:**
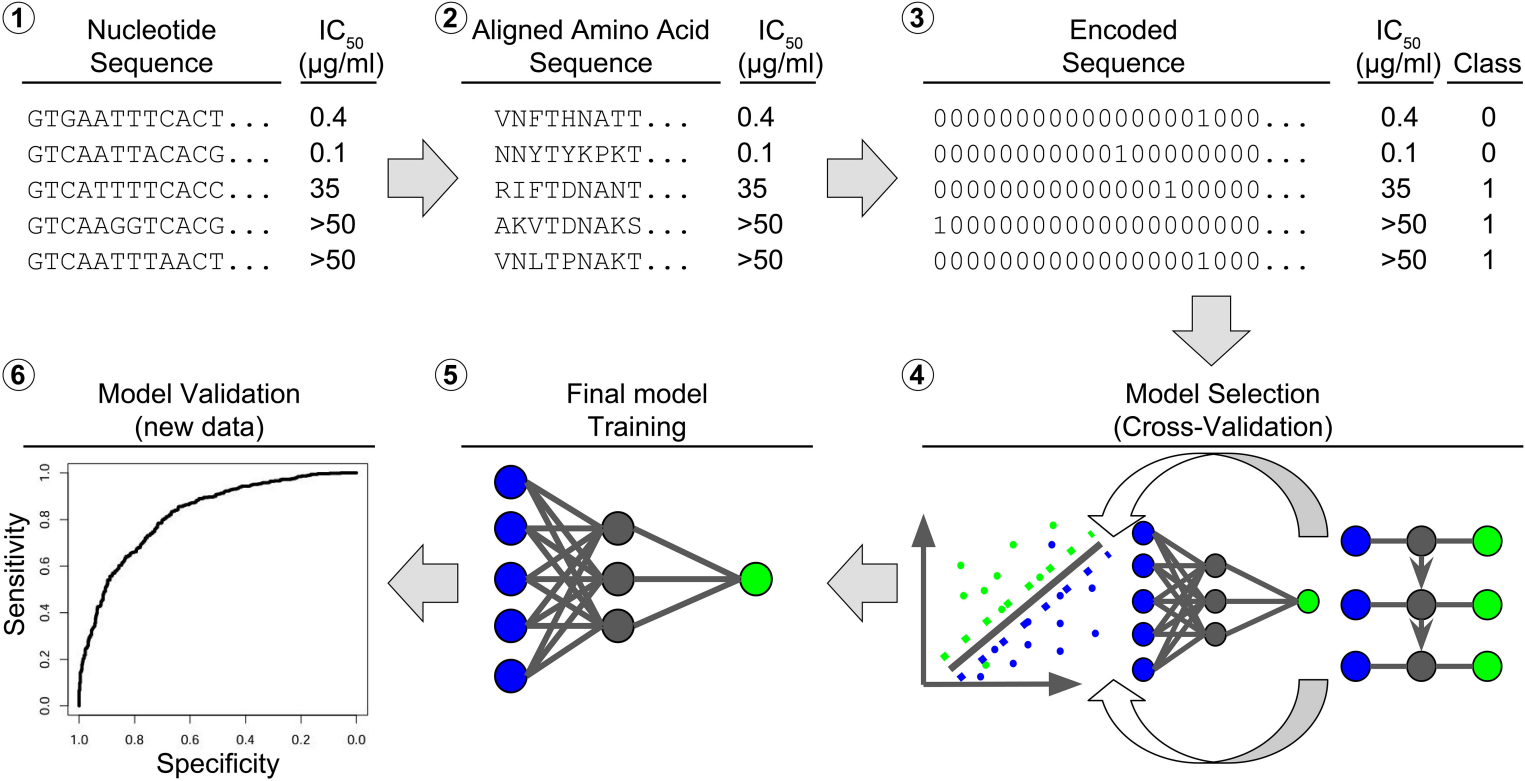
Bioinformatic workflow for model learning. The raw input consists of (1) nucleotide sequences of the ENV region, here the beginning of the D-loop part, paired with an IC value, e.g. IC50. The nucleotide sequences are aligned to a reference and (2) converted to amino acid sequences. The amino acid sequences are (3) converted to binary sequences by hot coding. The IC50 values are converted to binary class labels. The hot-coded sequences and class labels are (4) divided into a training set and a test set. The training set is used to rank different models by their cross-validation accuracy. The best model (5) is trained on the full training set and (6) validated on the test set.

While computational methods for HIV antibody research have recently been reviewed by Dănăilă et al. we focus here on the applicability of the different methods, their availability, and usability (103). We provide a brief description of the methods reviewed in **Table 1**, and a schematic overview of a machine learning pipeline for bNAbs is shown in **Figure 3**.

**Table 1:** Overview of reviewed approaches.

Reference	Method	Data	Complexity	Interpretability	Accuracy*
Gnanakaren et al., 2010 (104)	Rule-based	few amino acid positions	low	high	medium
Gnanakaren et al., 2010 (104)	logistic regression	few amino acid positions	medium	high	low to medium
Gnanakaren et al., 2010 (104)	ensemble of classification trees	all amino acid positions	medium	medium	low to medium
Hake and Pfeifer 2017 (126)	support vector machine	all amino acid positions	medium to high	medium to high	medium
Rawi et al., 2018 (128)	gradient boosting machine	all amino acid positions	high	medium	medium to high
Williamson et al., 2021 (129)	ensemble approach	all amino acid positions	high	medium	medium to high
Dănăilă and Buiu 2016 (136)	recurrent neural network (GRU)	all amino acid positions	high	low	medium to high
Igiraneza et al., 2023 (142)	recurrent neural network (LSTM)	all amino acid positions	high	low	medium to high

*Accuracy is not directly comparable due to different accuracy measures and differing amounts of data available.

## Rules for neutralization capacity of antibodies

One of the more computationally simple approaches is rule-based. In the case of bNAbs, Gnanakaran et al. have introduced a rules-based system for HIVAR and mutations on the HIV-1 envelope (104). Gnanakaran et al. used a signature consisting of seven positions of the viral proteome, and amino acids associated with susceptibility or resistance to antibody b12. If the signature includes at least four amino acids associated with susceptibility and at most one amino acid defined as resistance-associated, the virus is predicted to be sensitive to b12 and otherwise resistant. The signatures were identified by phylogenetic analysis of sequences and by identifying positions where mutated amino acids are enriched in resistant sequences. Other studies have also focused on the computational and non-computational identification of amino acid residues at mutated positions that predict sensitivity to antibody neutralization (105–108).

A rule-based algorithm can be complex involving many mutations and containing sophisticated rules that take into account the interactions of multiple mutations. For example, the Stanford algorithm mentioned above includes additional scores for combinations of up to four different mutations. Such complex rules do not currently exist for antibody resistance prediction.

## Machine learning for HIVAR prediction

Machine learning approaches to HIVAR prediction build predictive statistical models directly from data, avoiding the manual process of designing rules. Despite their simplicity, rule-based algorithms can still be quite predictive and in some situations more accurate than machine learning methods such as logistic regression and decision trees (104). In the Gnanakaran et al. study, the logistic regression approach replaced the binary influence of the signature positions with a continuous weight to more accurately account for the influence of each position on the prediction. A straightforward extension of a rule-based algorithm to a subset of mutations leads to a method that includes all positions in envelope region. Defining rules for all 857 amino acid positions of the envelope protein and possible mutations is difficult. Not enough is known about each individual position. We also do not assume that every mutation is relevant to the prediction, or rather that the contribution of the mutation may be very small but still relevant in some cases. A more refined approach is needed to handle this situation. In a third approach Gnanakaran et al. used decision trees considering all sequence positions combined with subsampling of the training set to avoid overfitting. Sensitivity or resistance was predicted by majority voting over all trees. Possibly due to the structure and relatively small size of the training and test sets, 251 and 56, respectively, logistic regression and the decision trees did not generalize as well as the rule-based approach.

## Analogy to previous drug resistance prediction

The use of alternative state-of-the-art machine learning algorithms is becoming increasingly relevant, especially since the amount of data for this problem has increased significantly since 2010. Machine learning algorithms have already been successfully implemented for HIV drug resistance prediction, for example in the geno2pheno system (https://geno2pheno.org/) (48). The procedure is to use available data and train a prediction model. More specifically, the model is trained on a set of viral genome sequences annotated as either susceptible or resistant (to a specific drug) with the goal of classifying a new sequence as either susceptible or resistant. No manual intervention is required during training, unlike with the rule-based approach and the logistic regression used by Gnanakaran et al., which are restricted to a subset of preselected mutations.

## Data availability and processing

Usually, one of the critical issues is the availability of annotated sequences. A large collection of sequences and their respective 50% inhibitory antibody concentration (IC_50_) and 80% inhibitory antibody concentration (IC_80_) values is available from CATNAP [Compile, Analyze and Tally NAb Panels (109)]. This database contains 162,679 neutralization assays for 2075 viruses (only 1846 viruses with matching envelope sequences) and 966 antibodies (619 heavy chain and 593 light chain sequences). IC_50_ and IC_80_ values are available for 158,640 and 68,028 assays, respectively (accessed January 2024). CATNAP is therefore a very important source of annotated sequence and neutralization data. However, even in CATNAP, data for some of the most recently discovered antibodies are still rare. Furthermore, it should be noted that most of the models reviewed here are trained on data available through the CATNAP database as there is currently no other established source or comparable dataset that allows the training of novel prediction models and machines. The CATNAP database also provides several in-house tools for analysis. The most basic analysis allows the user to individually select antibodies and envelope sequences contained in CATNAP, which are then visualized, described and summarized. For each antibody and sequence, the corresponding IC values are displayed, encoded by their amino acids, and color-coded by their degree of resistance. The user can also select the data by studies/publications. The same analysis is available for user-provided input (antibody, IC value, sequence). In a hybrid analysis, the user-provided data is put into context with the data from CATNAP. In addition, multiple criteria can be used to select specific antibodies. Criteria include neutralization strength and breadth, virus subtype, and the antibody binding regio. The result lists all antibodies relevant to the regarding input criteria.

A recently developed alternative to CATNAP is the HIResist database (110). HIResist uses CATNAP as a resource, among others, e.g., GenBank (109, 111). HIResist currently provides two computational tools that allow the user to search the database and select specific criteria for the antibodies. The results present the virus panel color-coded in resistant and sensitive with the corresponding IC value and amino acid sequence. Alternatively, the user can select a virus strain and obtain information on which antibodies the viral strain is resistant and sensitive to, again for a selected criterion, e.g., IC threshold. A third tool of HIResist provides insight into cross-sensitivities for user-selected pairs of antibodies. Again, the user selects thresholds and the tools visualize the sensitivity of the viral strains to both antibodies in a scatter plot. This allows the user to identify viral strains that are resistant or sensitive to both antibodies, or resistant to one and sensitive to the other.

The most common way to represent a viral sequence is the one-hot (or orthogonal) encoding used for categorical variables. For example, position 1 of the sequence is represented by 20 binary variables or features, one for each amino acid (112). Each variable indicates whether the respective amino acid or feature is observed at the position (1) or not (0) (**Table 2**). This theoretically extends the 857 amino acid long envelope to 17,140 features. However, not all amino acids are observed at all positions. In practice, depending on the antibodies and available sequences, we calculated that between 2500 and 7000 features are needed. This depends on the antibody and the matching viruses, i.e., if an amino acid at a position never occurs in any virus or the amino acid at a specific position is the same for all viruses, these features can be excluded without losing information. In addition to mutations, insertions and deletions can also be included as features. A simpler alternative to one-hot encoding is to code each position of the sequence that does not match a reference sequence as 1 and otherwise as 0, i.e., to code only the presence and absence of mutations, including insertions and deletions, without identifying the residue. The choice of reference is of secondary importance, since it is only used to compare the virus sequences. For example, suppose that reference A has amino acid X at position *p*, but reference *B* has *Y* at position *p*. Suppose further that half of the viruses have *X* at position *p* and the other half have *Y*. In this case it is irrelevant whether we use reference *A* or *B*. It may be relevant if we use reference *C* with *Z* at position *p*, because the two virus populations would be indistinguishable if we encoded only for the presence of a mutation and not for the specific amino acid. This type of encoding is called mutational encoding. The loss of information caused by mutational encoding can be avoided by using one-hot encoding for all positions and amino acids including wild type and all possible mutations. Thus, mutational encoding reduces model complexity at the cost of information loss. Another advantage of one-hot encoding over this simpler alternative is that the data can be represented as a sparse matrix due to the abundance of zeros. This can lead to significant improvements in efficiency (113).

**Table 2:** Example for one-hot encoding.

	A	R	N	…	M	…	W	Y	V
1M:	0	0	0	…	1	…	0	0	0
1Y	0	0	0	…	0	…	0	1	0

The first row encodes the first position of the envelope sequence of the HXB2 reference (Human immunodeficiency virus type 1 (HXB2), complete genome; HIV1/HTLV - Nucleotide - NCBI (nih.gov)). The amino acid at position 1 is Methionine (M) denoted by 1 at column M and 0 in all other columns. The second row encodes a potential sequence with a mutation leading to Tyrosine (M1Y).

Embedding is a technique to reduce the dimensionality of the sequence input while trying to minimize information loss (114). Methods used in text mining are transferred to amino acid sequence encoding. First, the amino acid sequence or document is slit into *k* sets or words each containing non-overlapping *k*-mers, e.g. AMINQACID is split into AMI,NQA,CID, and MIN,QAC, and INQ,ACI (3 sets of 3-mers). Methods like doc2vec use neural networks to learn a numerical vector with continuous values for each sequence, which captures the similarity of the sequences (documents) based on their *k*-mer representation (115). For example., AMI,NQA,CID, and MIN,QAC, and INQ,ACI are transformed into a vector like [0.1,0.5,0.2]. This can drastically reduce the dimensionality, especially compared to one-hot encoding. However, embedded sequences suffer from a loss of feature information. Instead of a feature like 1M, Methionine at position one, we have abstract features that are harder to interpret and harder to relate to the original input. Improved approaches have been proposed to address this drawback (116).

A third alternative for encoding amino acid sequences is to use a set of seven different interpretable features that are unique to each amino acid: steric parameter, polarizability, volume, hydrophobicity, isoelectric point, helix probability and sheet probability. I.e., each amino acid position is extended to seven continuous values (117). Meiler et al. train a symmetric neural network (autoencoder) on this seven-parameters-per-position encoding to further reduce the dimensionality (118). The autoencoder transforms the n-dimensional raw data into an m-dimensional embedding (*m* << *n*). This embedding is optimized by learning to retrodict the input data from the embedding.

All of these methods are limited by the fact that ENV is a highly mutated region of the HIV-1 genome (119). Especially, when compared to regions of interest for drug resistance (PR, RT, IN), the increased mutation rate of the ENV region poses an increased challenge for alignment prior to any amino acid sequence processing (120). In addition to the higher average number of mutations, insertions and deletions are common (121). In contrast, insertions and deletions in PR, RT, and IN regions are generally not observed. In practice, this could lead to entire regions being marked as low quality, and subsequently discarded, due to a high mutation rate.

## Support vector machines for resistance prediction

**Figure 4:**
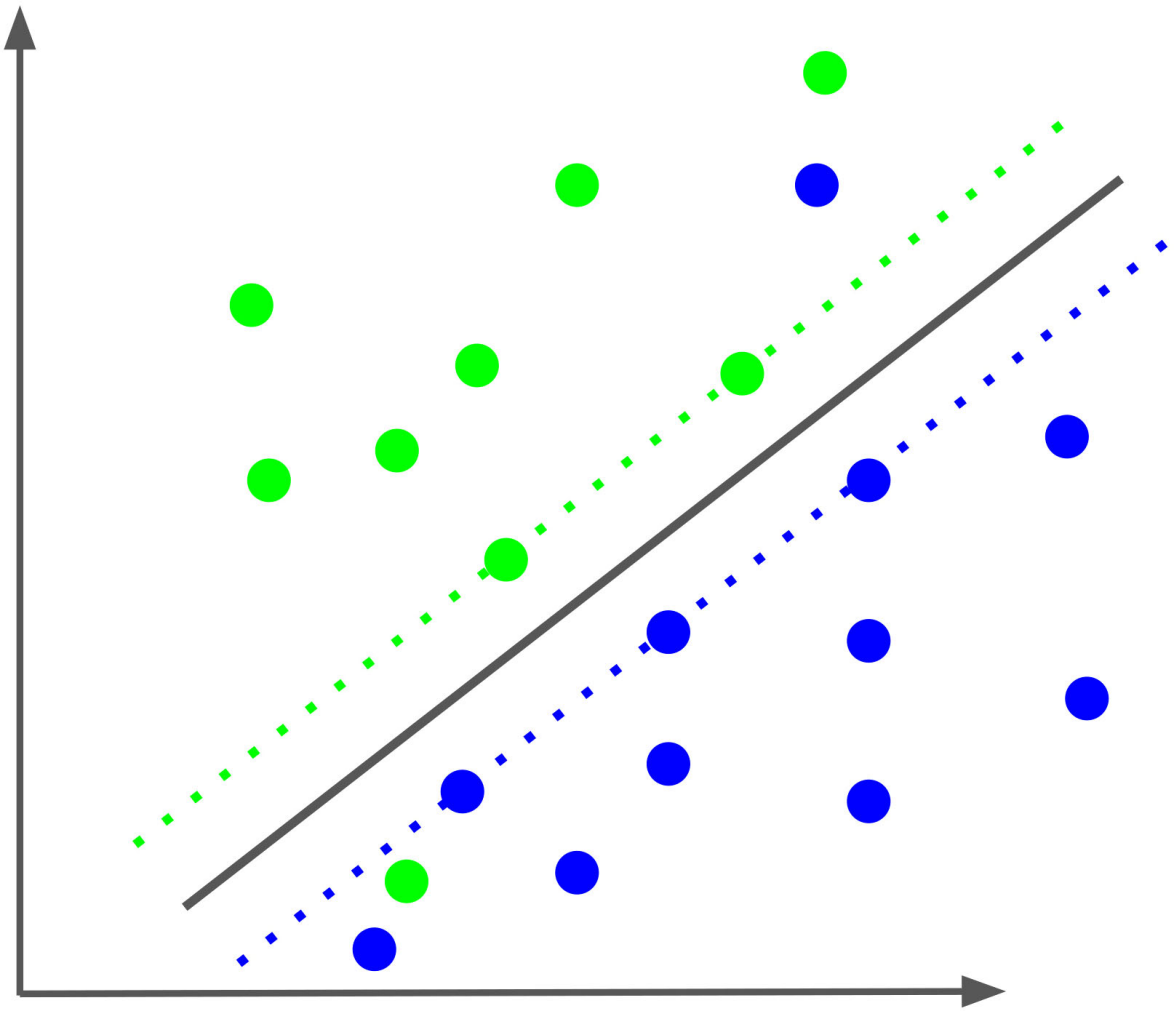
Schematic of a soft-margin Support vector classification (SVC). The SVC optimizes the separation of the two classes, colored green and blue, by a hyperplane, here projected in two dimensions. The two dotted lines represent the margin of separation for each class. The samples along the dotted lines and the two samples on the “wrong” side of the hyperplane are the support vectors that define the separating plane.

Support vector machines [SVM (122)] are a state-of-the-art method used for classification, in this case into two classes of sensitive and resistant virus variants, respectively, based on the amino acid sequence of the envelope protein. Each sequence or sample is represented by a vector of numbers, in our case about 2500 to 7000 of them. These are called *features*. In so-called *one-hot encoding* each feature represents an amino acid at a particular position in the sequence, and its value is 1 if we see that amino acid at that position and 0 otherwise. Each vector represents a point in a high-dimensional Euclidean space, where the number of numbers in the vector being the dimension of the space. Support vector classification (SVC) aims to divide the set of all points into two classes, in our case of sensitive and resistant samples, respectively, by a linear hyperplane, see **Figure 4** (here, the classes are indicated by the colors green and blue). In the SVM approach, we choose the (unique) hyperplane that has the greatest distance to the nearest data point. This distance is called the margin. The sample vectors closest to the hyperplane are called support vectors. In **Figure 4**, they are arranged along the dashed lines, both of which are at the margin distance of the margin to the hyperplane. The support vectors define the hyperplane in the sense that if you move one of them a little bit, the hyperplane will move, as well. Thus, these points are the only inputs that go into the linear equation that defines the separating hyperplane. New samples are then predicted based on which side of the hyperplane the points representing them are on, with each side representing one of the two classes. Typically, training data sets cannot be completely separated in this way, e.g., some points end up on the wrong side of the hyperplane. To deal with this problem, a modified so-called “soft-margin” support-vector machine (SVM) has been developed that allows for such violations but tries to minimize them. This also results in a larger set of support vectors (123). Training the SVM determines the hyperplane by calculating multiplicative weights for each feature, which are summed and added to an intercept value to compute the SVM score of the point. This score is equal to its (signed) distance from the hyperplane. The sign of the SVM score determines which side of the hyperplane the point is on and thus the class to which the point is assigned (sensitive or resistant). Alternatively, decision values can be transformed into probabilities for the class label (124), i.e., the probability that a virus is resistant to the antibody.

A characteristic element of SVMs is that not the data points themselves but only the inner product between pairs of them enter the equation of the hyperplane (125). Since the inner (dot) product can be understood as a measure of the similarity between the points in the pair, this allows for a powerful generalization of SVMs to nonlinear classification: Instead of the inner product, we can use other kernel functions that implement other notions of similarity between points, such as polynomial, radial, and sigmoid kernels (123). The dot product, called a linear kernel in this context, has the advantage that the feature weights are easily interpreted: The sign of the weight indicates which class the feature supports for the data point. The sign of the weight indicates the class the feature supports for the data point. The absolute value of the weight indicates its importance relative to other features. Especially in the case of binary features, a feature with twice weight of another feature is twice as important for the classification of the data point.

The only hyperparameter that needs to be either set or learned when training an SVM is a regularization parameter denoted by *C*. A large *C* will result in a better fit to the data used to train the model, which may come at the expense of the model’s ability to generalize, i.e, its classification accuracy on new data. In contrast, a smaller *C* can increase the generalization ability. *C* is typically optimized using cross validation.

Hake and Pfeifer used an SVM with an oligo kernel, which has already been introduced for sequence analysis (126, 127). This kernel can be interpreted as an extension of the linear kernel in two ways. First, the oligo kernel does not only compare single amino acids, but also *k*-mers, i.e. subsequences of length *k*. Second, the same *k*-mer does not only contribute to the similarity, if it is located at exactly the same position in both sequences. Rather, the oligo kernel still detects similarity, even if the *k*-mer is a few positions apart in both sequences. This allows for uncertainty, modeled by a Gaussian distribution, in regards to the position of the *k*-mer in both sequences. In addition to the penalty *C* the oligo kernel introduces *k* and the standard deviation of the Gaussian distribution as new hyperparameters to be optimized. Hake and Pfeifer evaluated the performance of the SVM with the oligo kernel by cross validating models learned from the CATNAP database. They trained one model for each antibody. Accuracy ranged from approximately 65% (35O22) to 84% (10–996) for eleven different antibodies targeting the V1/V2 loop, V3 loop, CD4bs and gp41-gp120. While the accuracy of the models for different antibodies showed considerable variation, the model for each individual antibody showed a prediction accuracy with little deviation.

## Ensemble approaches leverage several statistical models for prediction

Rawi et al. proposed to use an extension of the decision tree ensemble method called gradient boosting machine (GBM) and named their method bNAb-ReP (128). In this additive approach, the model is extended iteratively by modeling residual differences between the current model prediction and the response. A weak learner is added at each iteration. A regularization parameter is used to avoid overfitting. Rawi et al. make the final models more interpretable by computing feature importance scores. These scores correspond to the increase in prediction error after permuting the values of single feature. However, these feature importance scores are not part of the prediction model and do not directly convey a feature’s contribution to the prediction. Comparisons by Rawi et al. on test sets unrelated to the CATNAP database show higher performance of bNAb-ReP compared to Hake and Pfeifer’s oligo kernel. Rawi et al. did not compare the performance with Hake and Pfeifer on the CATNAP database. A comparison of the results in both articles would be compromised by the different dates of data access (2017 vs. 2019).

Ensemble approaches, such as the decision tree ensemble mentioned above, have the advantage of being less prone to overfitting as easily. In addition, instead of ensemble approaches based on subsampling that use essentially the same method each time, one can also use an ensemble of different methods to predict sensitivity to an antibody (129). Williamson et al. offer a software suite called *Super LeArner Prediction of NAb Panels* (SLAPNAP). SLAPNAP combines random forests (130), boosted regression trees (131) and the elastic net (132) into a single prediction model with cross-validated ensemble weights. Williamson et al. argue that it is not easy to identify the optimal model for each antibody. Therefore, the super-learner using multiple models is preferred (133). Williamson et al. encode the amino acid sequence indicating mutations, frameshifts, gaps, stops or sequons with respect to the reference HXB2. In addition, they encode the geographic region of origin of the respective sample (one-hot), the HIV-1 subtype (one-hot), the length and number of sequons, and the number of cysteines in different genomic regions, e.g., the envelope. For the classification task SLAPNAP’s performance is similar to bNAb-ReP, on average (81% vs. 84%). SLAPNAP is also designed to predict the continuous neutralization values (IC_50_ and IC_80_) (129). However, this task seems to be much more difficult, and the performance shows a high variance. Correlation values of 0.65 (IC_50_) and 0.52 (IC_80_) show at least a better performance than a random guessing approach. Williamson et al. argue that the lack of data, especially for IC_80_ values, is one reason for the relatively low performance. Of course, this could be improved in the future. SLAPNAP addresses the prediction of combinations by an additive or Bliss-Hill model (134).

## Neural networks for resistance prediction

**Figure 5:**
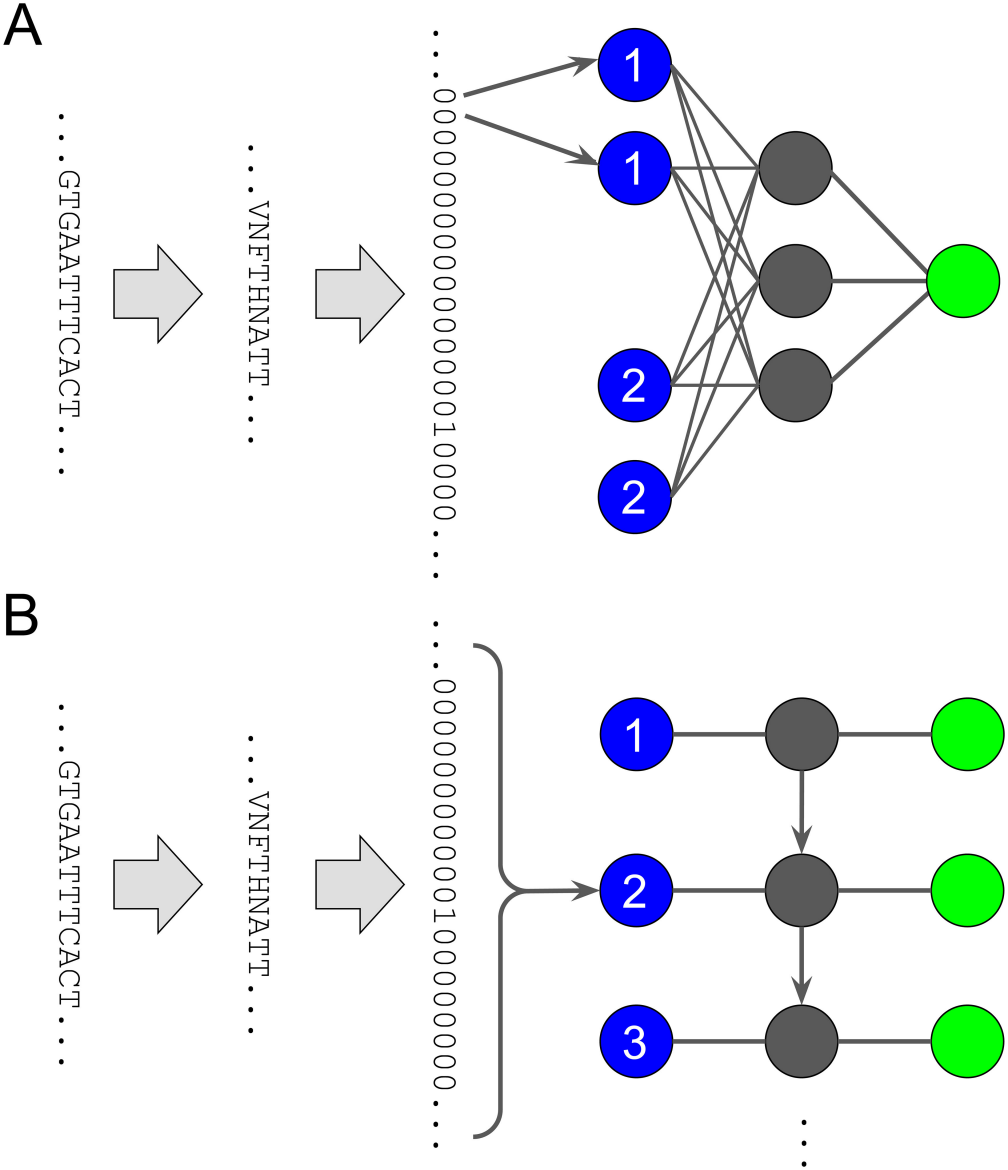
Schematic of small neural networks with only one hidden layer. **(A)** A neural network with a dense hidden layer of size three and a single output, as in binary classification or regression. Each input node corresponds to a position paired with an amino acid. For example, there are 20 input nodes for position one, one node for each amino acid. In this visualization, the first two amino acids at position one are different from V, so the first two inputs are 0. Only the 17th input is 1, because the 17th amino acid is V. **(B)** Schematic of an unrolled recurrent neural network. Inputs are shown in blue on the left, outputs are shown in green on the right. Hidden layers are shown in gray and are unrolled from top to bottom. At each time point or position, we feed 20 zeros and ones into the input node, one for each amino acid. In this visualization, the sequence shows amino acid N at position two, which means that only feature N, the 12th feature, in the one-hot encoded sequence is 1, while all others are 0. The weights for the (horizontal) edges from inputs to any hidden layer, the (vertical) edges from hidden layer to next hidden layer, and the (horizontal) edges from any hidden layer to outputs are the same for each position. In the case of binary classification or regression, the output layer would be another hidden layer connected by a dense layer to a single output.

With the ever-increasing amount of data, deep learning approaches such as neural networks (NNs) are a logical choice for modeling of antibody resistance prediction (103, 135, 136). NNs have an input layer and an output layer of computational units called neurons or nodes. Here the input layer receives the representation of the virus sequence and the output layer provides the resistance phenotype. Between the input and output layers are one or more additional so-called hidden layers of neurons (**Figure 5**). Each node calculates an output value from the values of inputs. These are provided by the outputs of the nodes in the previous layer (feed-forward). At each node, an affine function is applied to the input values, the result of which is then fed into a nonlinear activation function to produce the output value. Common activation functions are the sigmoid function, and the ReLU function, both of which project produced values in the range of [0,1]. Recurrent neural networks (RNNs) break the strict feed-forward mechanism and allow nodes in downstream hidden layers to be inputs of nodes in upstream hidden layers, allowing feedback loops (recurrent edges) (137). This is particularly relevant for longitudinal data, where recurrent edges facilitate the reuse of the same hidden layer multiple times to represent consecutive time points. Due to parameter sharing, model complexity is independent of the number of time points. Neural networks are trained using the gradient-descent method, which iteratively reduces an appropriate error function, also called a loss function. Providing recurrent edges can lead to the problem of exploding or vanishing gradients, which means that, for an error from a previous time point, the derivative of the loss function can reach zero or very large values exponentially fast. The actual behavior depends on the activation function and whether the absolute weight of the recurrent edge is less or greater than 1. Several methods like gated recurrent units (GRU) or Long Short-Term Memory (LSTM) have been introduced to prevent vanishing or exploding gradients (138–140). This is done by adding a more complex structure that is used to determine whether input from previous hidden states should be included in the current hidden state. LSTM are more effective than GRUs, but GRUs are easier to train.

Dănăilă and Buiu explored several bidirectional RNN configurations, but the inherent complexity of RNNs prevented them from an exhaustively exploring model and parameter combinations. Instead of learning the resistance from the virus sequence alone, they added the sequences of the antibody’s heavy and light chains as input. This has the advantage of learning a single model that covers all the antibodies, so that those with only few samples can benefit from the additional power of the other antibody-sequence pairs. The basic structure of each explored NN consists of two parts. The first is an antibody type prediction RNN. This NN learns an embedding for the antibody sequence (heavy and light chain) during training. The second part is the actual resistance prediction. The input is the virus sequence and the previously learned antibody embedding. The output in each NN is computed by a fully connected layer or dense layer, which means that all nodes from the previous hidden layer are connected to one node in the next layer, here the output. The dense output layer is combined with drop-out to reduce overfitting (141). In a layer with drop-out a percentage of edges is removed in each training iteration, i.e., all connections to and from a subset of the nodes are ignored. The final trained layer will be fully connected, but should be less prone to overtraining due to the randomization during training. RNNs have the problem that amino acids are considered more closely related if they are positionally close in the sequence. Only the hidden state of the last sequence position is included in the output. However, sometimes amino acids that are further apart improve the prediction when considered in context. Including attention in the RNN means that all hidden states of all previous positions are included in the next output. But before the output is produced, the hidden states are assigned weights, and these weights are normalized, usually using softmax to compute a probability distribution for the hidden states. In this way, the trained NN is able to pay more attention to relevant amino acids further away in the sequence. Overfitting is again controlled by drop-out learning. The hidden layers in the RNNs are bidirectionally connected. To avoid aforementioned gradient vanishing/exploding problem, the authors used GRUs in all recurrent layers. As an alternative to RNNs Dănăilă and Buiu also trained a transformer. A transformer is a NN with multi-head attention, which means that not only one weight vector is learned for all previous hidden states, but several. This makes the NN more flexible. In addition, the transformer includes position encoding because, unlike RNNs, the whole sequence is processed in parallel, which improves the training time, and without positional encoding the sequence information would be lost. As before the output is computed by a dense layer. For the comparison with other methods Dănăilă and Buiu fine-tuned their model to each antibody. To do this, they trained a model using all the samples without the specific antibody. They then used cross-validation to learn the hyperparameters based on the samples with the antibody. The RNN method using amino acid properties as input of Dănăilă and Buiu outperformed both bNAb-ReP (88% vs 84%) and SLAPNAP (83%), on average, but not for all antibodies. In particular, for antibodies targeting gp41 MPER, the gp41-gp120 interface, and fusion peptide bNAb-ReP and, to a lesser extent SLAPNAP outperform the RNN. Dănăilă and Buiu also investigated multi-task learning by predicting the class of the antibody together with the viral resistance, but observed no significantly different performance. The transformer also showed no significant improvement.

Similar performance was observed for RNNs using LSTM instead of GRU for the hidden layers (142). Igiraneza et al. train a language-based universal model (LBUM) to address two challenges. The first challenge is the potential influence of the virus subtype and the underrepresentation of some subtypes in the training data. This has only been partially addressed by including subtype as a covariate in the model (129). The second challenge is to predict of outcome of potential antibody combinations. On average LBUM performs similarly to GBMs and random forests with sometimes large differences between individual antibodies. Accuracy ranged from 59% (35O22) to 94% (DH270.1) (142). Similar to the previous RNNs of Dănăilă and Buiu the performance was lowest for the classes targeting gp120-gp41 and MPER. However, the ensemble approach, which combines all three methods by averaging the prediction probability, was the most accurate. Igiraneza et al. also showed that subtype bias is present in unbalanced data and that it can be partially corrected by using data on non-broadly neutralizing antibodies (non-bNAbs) by balancing the dataset. In contrast to Dănăilă and Buiu, Igiraneza et al. did not use the antibody sequences as input for each independent antibody model but learned a randomly initialized antibody vector based on neutralization data and virus sequences. This vector (or context) was intended to capture the resistance pattern for each antibody, i.e., a vector of continuous values encoding the resistance of the available viruses to the antibody. Igiraneza et al. showed that this pattern is similar for antibodies targeting the same epitope. This pattern showed that antibodies of similar classes clustered together. New antibodies were predicted with an accuracy of 72% by assigning the class of the closest antibody in the training set. Some antibodies did not cluster within their respective classes, which Igiraneza et al. could explain by different mechanisms in the action of the antibodies.

In summary, previous research has shown that relatively simple approaches, such as defining rules for resistance-associated mutations, can help to distinguish between sensitive and resistant viruses. The growing amount of data now allows the use of much more complex and precise machine learning algorithms to be employed (103, 126, 128, 129). Support vector machines, even with a linear kernel, are still competitive and have the added advantage of being interpretable (128). Algorithms using neural networks (103, 142) show high prediction accuracy which may increase in the future as more data becomes available. However, due to their complexity, such approaches need additional technology for to become interpretable (128). While the prediction accuracy of the leading classification methods is relatively high (around 80%), regression of the actual IC_50_ or IC_80_ values remains a greater challenge. In addition, the accuracy is highly dependent on the bNAb under consideration, reaching accuracies above 90% for some and some as low as 60% for others. In conclusion, there is a need for further research in this area.

## Animal models for detecting *de novo* HIVAR that emerge during treatment

Most *in vivo* data on the antiviral effects of bNAbs have been generated using HIV-1 infected humanized mice and non-human primates (NHPs) that have been infected with chimeric simian-human immunodeficiency virus (SHIV). Humanized mice that have received human cells, such as CD34+ human cord blood, are able to develop and maintain human B, T, and dendritic cells (143). Thus, they can be infected with replication-competent HIV-1 and maintain high viral loads for several weeks (28). To use NHPs as a model for HIV research, SHIV, a chimeric virus that contains elements of both simian and human immunodeficiency viruses, has been used and shown to result in sustained viremia in infected NHPs (29, 144).

While the *in vitro* models and assays described above can provide information on residues critical for neutralizing a specific virus, these models cannot assess the impact of specific mutations on the replication capacity of a virus that is also often referred to as ‘viral fitness’ (145). *In vivo*, however, the effect of a single mutation or a combination of mutations on the viral fitness is crucial because it determines the extent to which that particular virus will be part of the viral swarm (146). Thus, mutations that lead to a viral escape from the humoral immune pressure but compromise viral fitness may not be able to replicate sufficiently and may not be detectable anymore *in vivo* after several days or weeks of infection. However, the first *in vivo* experiments using single highly potent bNAbs for treatment in animal models clearly showed that escape mutations leading to viral resistance but allowing viral replication exist and emerge rapidly after treatment (28, 29, 144). The viral kinetics of rapid viral decline followed by a rebound viremia in these animal models are highly comparable to the kinetics found later in clinical trials with viremic patients. Moreover, sequencing of the rebounding viruses revealed escape mutations to the respective bNAbs at the same residues that were later found in patients that were treated with these monoclonal antibodies (28, 29, 147).

In summary, *in vivo* animal models provide a critical platform for studying HIV antibody resistance, providing insights into viral escape mechanisms and aiding in the development of effective interventions. While challenges remain, the continued refinement of humanized models and the integration of findings into clinical strategies hold promise for combating HIV antibody resistance. Combining *in vivo* studies with *in vitro* experiments and clinical observations offers a comprehensive approach to tackling the challenge of HIV antibody resistance.

## Escape mutations as detected during bNAb treatment in clinical studies

Clinical studies have shown that passive infusion of bNAbs as monotherapy can transiently suppress viremia. The CD4bs bNAb 3BNC117 as HIV-1 monotherapy effectively suppressed viremia in infected patients between 0.8 and 2.5 log_10_ for up to 28 days (36). Since this first clinical study, many bNAbs, targeting different epitopes, have been applied to viremic individuals, to patients before or during an analytical treatment interruption or to healthy individuals for passive protection (148). In all of these scenarios, one of the major hurdles for bNAb application to humans were pre-existing bNAb resistance mutations or *de novo* viral escape that developed during treatment (81).

This was first observed in trials using a single bNAb. Although 3BNC117 monotherapy was effective in suppressing viremia during weeks 1 and 2 of treatment, rebound was observed in all patients even though 3BNC117 was still present in the participants’ plasma at sufficient levels. In this study, the majority of individuals treated with higher doses were pre-screened for 3BNC117 sensitivity using patient PBMC viral outgrowth assays (VOA). All patients with 3BNC117-sensitive viral outgrowth cultures showed a significant decrease in viremia after bNAb treatment. However, among the non-screened patients, one individual did not respond to 3BNC117 treatment and was later found to be completely resistant to this bNAb. Data evaluating the efficacy of a single infusion of VRC01 in suppressing HIV-1 infection showed a similar pattern of rapid development of escape mutations at sites that were expected to be critical for VRC01 neutralization (96). Following up on the CD4-binding-site bNAbs clinical studies, a rapid development of escape mutations was also observed upon a single administration of bNAbs targeting the V3-glycan. 10-1074 and PGT121 also lead to a viral transient suppression of viremia in patients with a rapid viral rebound after about 2 weeks (35, 149).

As shown earlier with classical antiretroviral treatment (ART), combinations of drugs can establish long-term control of viremia in people living with HIV (150, 151). And indeed, double administration of bNAbs targeting two different epitopes on the HIV-1 envelope trimer, 3BNC117 and 10-1074, demonstrated a longer suppression of viremia (37). Among the four subjects harboring viruses susceptible to dual antibodies, immunotherapy resulted in a remarkable average reduction of viremia of 2.05 log^10^ copies/ml. This reduction was sustained for up to three months after first infusion. However, while none of these individuals developed resistance to both antibodies, *de novo* escape mutations to either one of the applied antibodies were found in the rebounding viruses. Most novel escape mutations were observed for antibody 10-1074, due to its longer half-life and a *de facto* monotherapy at the end of the study, while antibody 3BNC117 was observed subtherapeutic levels. Thus, this study also showed that screening of patients via viral outgrowth assays might not be feasible for future studies, as this method missed pre-existing 3BNC117 or 10-1074 resistant strains in several patients that were later found via single genome analysis from pre-infusion samples (37). Pre-screening for bNAbs-resistant viruses in patients may be critical for broader clinical use of bNAbs in the future, as shown in the first triple bNAb treatment study (152). When a combination of VRC07-523LS, PGDM1400, and PGT121 was used to treat viremic individuals, it was able to reduce an HIV RNA by a maximum mean of 2.04 log^10^ copies per ml. However, similar to the previous single bNAb treatment studies, a rapid rebound was observed in the majority of patients. *Post-hoc* analyses showed that 2 out of 4 of the viremic patients treated with triple therapy already had circulating strains that were resistant to one or two bNAbs, resulting in only one or two active bNAbs. In addition, the other patients who were sensitive to all bNAbs quickly developed a *de novo* escape to the majority of the antibodies used (152).

Pre-existing resistant strains were also a major problem in several analytical treatment interruption (ATI) studies, which evaluated the effect of bNAb treatment on the time to viral rebound after ART discontinuation. Several studies showed that patients who received bNAbs during or before an analytical treatment interruption rebounded faster if their pre-existing strains were resistant to the single or combined antibody used (38, 153–156).

In addition, these studies showed how difficult it can be to screen patients on ART for pre-existing resistant viral strains. In viremic patients, analyses of the circulating strains can easily provide information about existing resistance, as large proportions of circulating strains are replication competent (157). In contrast, the vast majority of viruses in the viral reservoir of long-term treated individuals are defective and thus, single genome analyses of the envelope genome may detect resistant or sensitive strains that could belong to non-replicative clones and are thus are not part of the rebounding viral swarms (158). On the other hand, viral outgrowth cultures underestimate the diversity of the viral reservoir (91).

Overall, these clinical trials have limitations to thoroughly investigate HIVARs, given the small sample size of participants and the different methods used to pre-screen individuals for their *de novo* or pre-existing bNAb resistance mutations.

For future clinical trials, and especially for wider clinical use, it would be important to develop a robust assay that can assess the frequency of specific mutations to each bNAb and the resulting HIVARs. There is a need to evaluate HIVARs in large cohort studies consisting of different HIV clades to get a better idea of the frequencies of resistant patients to each bNAb and to better design future bNAb combination trials. The large pseudovirus panels currently used to assess the potency and breadth of a particular bNAb are mostly composed of sequences isolated more than two decades ago (159, 160).

In conclusion, clinical trials testing single or combined bNAbs in viremic patients or in patients undergoing ATI may reveal novel escape mechanisms of HIV-1 that have not been predicted by other *in silico*, *in vitro* or *in vivo* models. In addition, these studies demonstrate how critical it will be for the future clinical use of bNAbs to predict a patient’s bNAb sensitivity with accurate methods that are rapid enough to be incorporated into daily practice (37, 153, 154).

## Antibody resistance in HIV-1 cell-to-cell transmission

HIV-1 transmission can occur by infection of cells by free viral particles or by interaction of infected cells with neighboring healthy cells. Despite very high bNAb titers in the serum of bNAb-treated patients, antibody resistance can develop due to the rapid transfer of viral particles from one cell to another. Cell-to-cell transmission spreads the virus faster than free-circulating viruses in the host (161–163), and may be a primary driver of immune evasion and establishment of the latent reservoir in memory T cells (164). HIV-1 reactive bNAbs prevent viral cell-to-cell transmission by interfering with the fusion of infected and healthy target cells, thereby intercepting the transfer of viral particles (165). How quickly HIV env transitions from CD4 engagement to coreceptor binding on target cells determines the neutralizing activities of bNAbs (166, 167). Thus, understanding the neutralizing breadth and potency of bNAbs during the cell-cell transmission mode of a diverse HIV strain is an important approach to determine novel immune evasion pathways.

Several studies have accessed the efficacy of bNAb in the cell-cell transmission mode (168–170). However, these studies failed to accurately compare bNAb activities in cell-cell transmission mode and free virus in the same experimental setting. Abela et al. developed a more direct experimental approach to quantify free virus and cell-cell transmission in a single setting (167). The authors used a luciferase-tagged TZMbl cell line system in which free virus released upon co-culture with JR-FL-infected PBMC does not infect TZMbl cells in the absence of DEAE dextran. This approach allows cell-cell transmission without TZMbl infection by free virus. However, this assay is limited to the study of cell-cell transmission using engineered target cell lines.

Based on this model, studies have shown that CD4bs bNAbs that are highly effective in neutralizing free virus spread may be less effective during cell-to-cell transmission (167, 171). Further work using the cell-cell transmission assay has shown that bNabs targeting the V1/V2 (PGT145, PG16) and V3 loops (PGT121, PG128, 2G12) retain their potency against free viruses during cell-to-cell transmission. A slightly reduced neutralizing activity for MPER-targeting bNAbs during cell-mediated transmission has also been reported (167). One explanation for the resistance of CD4bs bNAbs in the cell-to-cell transmission mode is that Env crosslinks with CD4 with high avidity and thus requires a high amount of neutralizing antibodies to disrupt this complex (172). In addition, the use of the cell-cell transfer system has informed on the vital impact of scFV in neutralizing viruses in cell-free and cell-cell transfer modes (173).

In conclusion, the use of cell-cell transmission assay will be beneficial in designing bNAb combination therapies or engineering potent multispecific antibodies with bNAbs targeting different HIV transmission modes.

## The importance of detection of HIVAR in the reservoir

HIV latency is established when infected long-lived CD4^+^ T cells enter a dormant state (174). The HIV latent reservoir develops at the early stage of infection (175, 176), with a half-life of the participating proviruses of about four years (177, 178). However, the field continues to develop new assays to detect, measure and characterize the latent HIV reservoir in people living with HIV. Even the most advanced assays can vary by several logs in their estimates of the reservoir size of infected CD4 cells per million CD4 cells (179). In addition, the vast majority of proviruses are defective and thus unable to produce replication-competent viruses (158, 174, 180, 181). It is well known that 97% of latently infected CD4^+^ T cells in the HIV reservoir have defective integrated proviruses characterized by hypermutation (GG → AG or GA → AA) and large deletions, with only about 3% of the proviruses being intact (158, 181–184).

Most available assays for measuring the size of the HIV reservoir are limited to proviruses in blood samples. However, it is widely recognized that human tissues serve as an HIV reservoir, and proviruses in these tissues are quite different from circulating proviruses (185). These hurdles make it difficult to develop assays that not only accurately determine the size of the reservoir but also provide single-genome sequence information about the identified replication-competent proviruses. Sequence information on these proviruses would be important for detecting HIVAR in the HIV reservoir in order to better design and plan bNAb therapy and prevention approaches. While there are some methods that can obtain envelope genome sequences from complete and intact HIV-1 genomes, most of these assays are very expensive, labor-intensive and time-consuming and are thus not feasible for larger clinical trials or a wider clinical use (180, 186).

PCR-based assays are the most commonly used technique for measuring total HIV DNA. These assays measure the number of copies and sequence information of proviral DNA in the HIV reservoir using small samples, thus providing ‘surface level’ insight into HIV-1 latency in a less laborious, rapid, and highly cost-effective approach. However, most proviral DNA detected by conventional PCR of specific HIV-1 genome segments belongs to defective proviruses (158, 180, 184, 186). Most clinical trials to date have used the ‘Phenosense’ assay developed by Monogram Biosciences to screen patients for their bNAb susceptibility (187, 188). For this assay bulk envelope amplicons from bulk CD4-cells are cloned into reporter-pseudoviruses, which are then tested for their sensibility to specific bNAbs. Thus, this method does not discriminate between envelopes from intact and defective proviruses, making it likely that the vast majority of envelopes tested are from defective envelopes. However, when the sensitivity results of this assay were compared with, for example viral outgrowth assays or genotypic assays, the results of the PhenoSense assays and VOAs were comparable in terms of the bNAb sensitivity of the patient samples tested (189, 190). Thus, although this assay may phenotypically describe the bNAb sensitivity of envelopes derived mostly from defective proviruses, its prediction is mostly consistent with that assessed during VOAs. Whether such predictions are also accurate enough for a later widespread clinical use of bNAbs, e.g., during analytical treatment interruption, remains to be determined in larger clinical trials. Several groups have developed assays to detect intact proviruses from full-length sequencing of proviral strains, which could be used to better predict bNAb sensitivity from intact proviruses (180, 181, 186).

One of the most accurate techniques for determining the infectious units per million (IUPM) of the replication-competent proviruses in the HIV reservoir is the quantitative viral outgrowth assay (QVOA). In this assay, latently infected CD4^+^ T cells derived from HIV-1-positive patients are serially diluted *in vitro* and stimulated to reverse latency and initiate the release of infectious virions to infect CD4^+^ T cells from uninfected donors or cell lines such as SUPT1/CCR5 or MOLT-4/CCR5. While most PCR-based methods described above tend to overestimate the IUPM of the reservoir, the QVOA (191, 192) and its variants (193–195) might underestimate the size of the reservoir. This is because not all replication-competent proviruses can be induced with a single round of stimulation or are able to replicate under the specific *in vitro* conditions required for detection (158). In addition, the outgrowth viral strains may have poor viral fitness *in vivo* and could thus may be clinically irrelevant for future clinical trials. Although this assay is laborious, time-consuming (14 - 21 days), and requires large amounts of PBMCs, it is one of the few assays that not only detects intact proviruses, but also results in cultures of replication-competent viruses that can be used directly for phenotypic assays in downstream analyses (191, 192, 196, 197). This feature was used by Lorenzi et al. who developed a qualitative and quantitative viral outgrowth assay (called ‘Q^2^VOA’). Here, the QVOA protocol was optimized to increase the likelihood of identifying a large number of individual replication-competent proviruses in the reservoir and to study their genetic and phenotypic diversity. Replication-competent proviruses isolated by Q^2^VOA can be further tested for their neutralization sensitivity or resistance to bNAbs *in vitro* (91, 198).Thus, this assay may be the most accurate assessment to date of a patient’s bNAb sensitivity to replication-competent quasispecies in the viral reservoir to date. However, it is also time-consuming, extremely costly, laborious, and requires large amounts of PBMCs that often require a leukapheresis (91). In addition to these *in vitro* VOAs, mouse models have also demonstrated their utility in identifying replication-competent proviruses through *in vivo* outgrowth assays. In an adoptive transfer study, researchers transferred PBMCs from SIV-infected monkeys and HIV-infected patients on ART into NSG mice, resulting in measurable viral loads in the recipient mice after 1-2 weeks (199). Future studies could use the outgrown viruses to test bNAb sensitivity in downstream assays.

In conclusion, measuring the replication-competent reservoir for its bNAb susceptibility will be a key factor for future cure trials and for future clinical use, such as transitioning patients from effective daily ART to long-term bNAb treatment. While several different and unique methods could be used to test for HIV-1 antibody resistance in the viral reservoir, no method is yet available that can accurately determine the bNAb sensitivity of the viral reservoir with a small number of PBMCs and a short turnaround time. Thus, while bNAbs have promising properties and are likely to become part of the regular arsenal against HIV-1, novel assays need to be developed that can test the viral reservoir for HIVAR in a timely manner and through regular blood draws in routine clinical care.

## Discussion

HIV-1 reactive bNAbs are promising novel agents for the treatment and prevention of HIV infection. Moreover, with their FC-mediated effector functions, they could be part of novel strategies leading to functional cure of HIV-1 infections (81). However, although bNAbs have several advantages over classical antiretroviral therapies, they also have critical disadvantages. Among other things, such as the need for subcutaneous or intravenous injections to deliver bNAbs, their more complicated storage due to the need for refrigeration, and their relatively high production costs, one of the main hurdles to wider use of bNAbs in the clinic is the occurrence of HIVAR (200). Unlike antiretroviral therapy (ART), which is effective against nearly all strains and only loses its activity with the emergence of resistance mutations that develop in patients on sub-therapeutic levels of ART (often due to factors such as non-adherence), broadly neutralizing antibodies (bNAbs) often struggle to neutralize specific strains. This leads to scenarios where multiple bNAb regimens functionally act as monotherapies if only one of the administered antibodies can effectively neutralize the virus in the patient, resulting in the rapid emergence of viral variants that can escape monotherapy (152).

**Figure 6:**
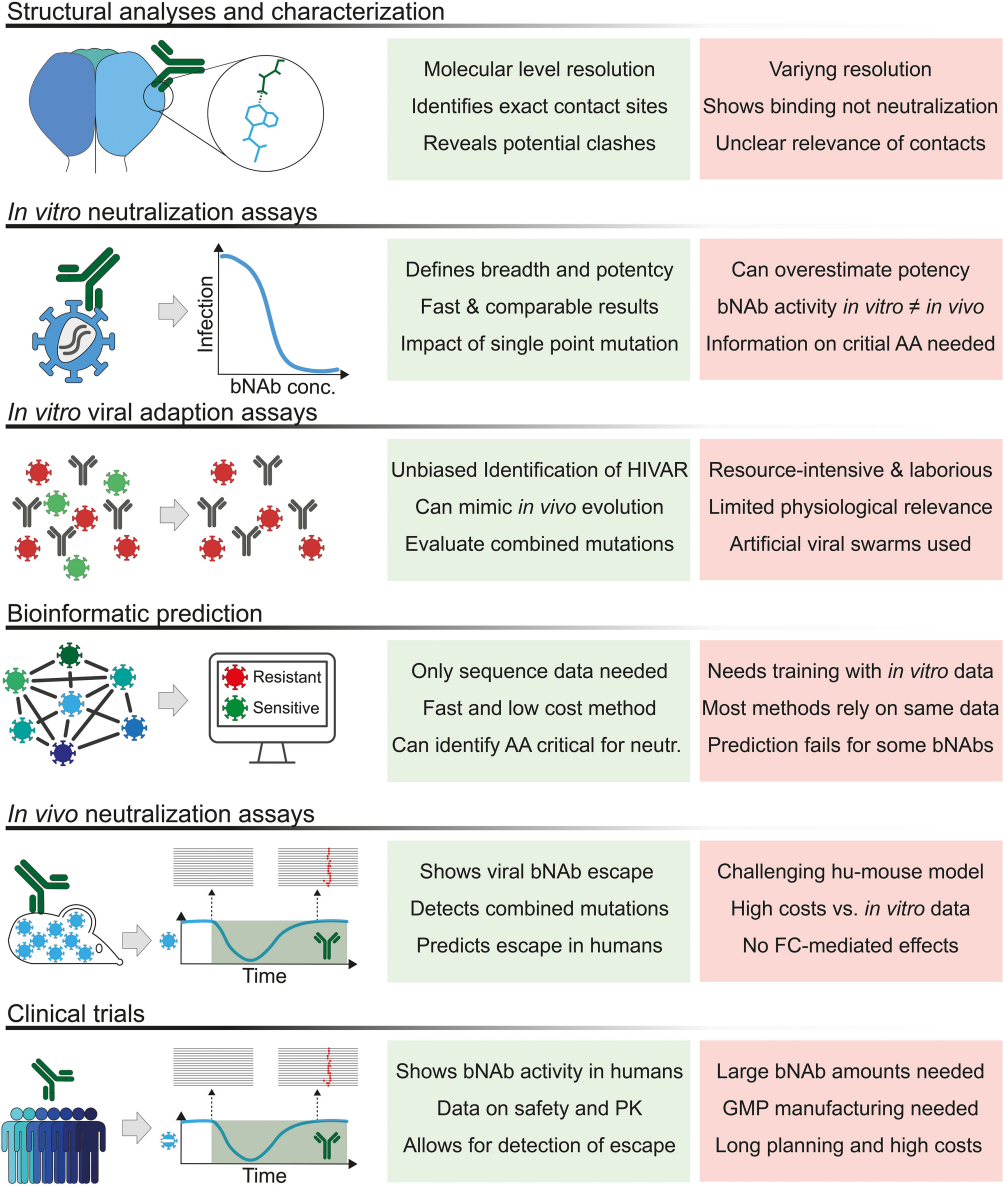
Approaches and methods for detecting and assessing HIV-1 antibody resistance. Overview of the various approaches, assays and methods currently used to detect, describe and characterize HIV-1 antibody resistance (HIVAR). AA, amino acid; bNAb, broadly neutralizing antibody; conc, concentration; GMP, good manufacturing practice; PK, pharmacokinetics.

Thus, the identification and understanding of HIVAR is critical for all future clinical applications of bNAbs for the treatment and prevention of HIV-1. As summarized above, a variety of methods and approaches are already available for detection and characterization of HIVAR. Structural data on envelope-antibody interactions can provide a first indication of residues that may affect sensitivity to a given antibody. *In vitro* data from pseudoviruses can reveal residues critical for neutralization. Large datasets of annotated sequences with neutralization profiles can be used to train machine-learning approaches that can predict bNAb sensitivity based on envelope sequence data. Finally, sequencing of rebound viruses from *in vivo* data from viremic animal models or from clinical trials treating viremic individuals can be used to detect viral escape variants that emerge after multiple rounds of viral replication (**Figure 6**).

Despite this wide variety of different methods, none of them is feasible for daily clinical use, for example to screen patients for HIVAR before starting long-term treatment with bNAbs. Most of the methods used and described here are either too inaccurate, too laborious, too expensive, or too time-consuming. Therefore, new approaches to characterize and identify HIVAR in patients are urgently needed to take full advantage of the novel features that bNAbs bring to the treatment and prevention of HIV-1.
